# Socio-Economic Impact of and Adaptation to Extreme Heat and Cold of Farmers in the Food Bowl of Nepal

**DOI:** 10.3390/ijerph16091578

**Published:** 2019-05-06

**Authors:** Nanda Kaji Budhathoki, Kerstin K. Zander

**Affiliations:** 1 Northern Institute, Charles Darwin University, Ellengowan Drive, Brinkin 0909, Darwin NT, Australia; kerstin.zander@cdu.edu.au; 2 German Development Institute, 53113 Bonn, Germany

**Keywords:** climate change, cold spells, crop production, heat waves, public health, labour productivity loss

## Abstract

Farmers worldwide have to deal with increasing climate variability and weather extremes. Most of the previous research has focused on impacts on agricultural production, but little is known about the related social and economic impacts on farmers. In this study, we investigated the social and economic impact of extreme weather events (EWE) on farmers in Nepal, and explored how they coped with and adapted to heat waves and cold spells between 2012 and 2017. To address these aims, we conducted a survey of 350 farms randomly selected from the Bardiya and Banke districts of the Terai lowlands of Nepal. They were specifically asked to rate the impacts of extreme temperatures, as well as their effect on labour productivity and collective farmer health, and the detailed preventative measures they had implemented. About 84% of the farmers self-reported moderate or severe heat stress during the last five years, and about 85%, moderate or severe cold stress. Likewise, the majority of respondents reported that both farmer health and labour productivity had been compromised by EWEs. Productivity loss had a strong association with the perceived levels of heat and cold stress, which, in turn, were more likely to be reported by farmers with previous EWE experience. Potentially due to the increased care required during EWEs, those farmers with livestock reported increased heat and cold stress, as, surprisingly, did those who had implemented adaptation measures. Farmers seemed to be less prepared for potential threats of cold spells than heat waves, and therefore less likely to adopt coping strategies, since these are a recent phenomenon. This study identified some limitations. The cross sectional and self-reported data, as a common source of information to estimate health impact, level of heat/cold stress and labour productivity loss. Community-based education/community engagement programs could be developed to facilitate proactive adaptation.

## 1. Introduction

Leading to rising temperatures and increasing climate variability, including more frequent and severe extreme weather events (EWE) [1], the global impacts of climate change on agricultural and food systems are substantial, putting food security and the livelihoods of many at risk [2,3]. As the climate becomes more volatile, some parts of the world are projected to be profoundly affected by the intensity of extreme cold events, which are expected to persist late into the 21st century [4]. Climate-change-related extreme events impose substantial economic and social burdens to global society [5], particularly in developing countries [6]. Consequently, to reduce the social and economic burden, it is essential to understand how weather or climate, as well as social and economic factors, interact to influence the nature and implications of climate impacts, and to identify adaptation gaps and implement cost-effective strategies [5].

While there are many studies on climate change impacts, such as the impact of floods on health [7,8], there is still little research on the social (health) and economic (labour productivity loss) effects of extreme temperature in farming communities. Rather, studies have focused on the damage from severe disasters such as floods and tsunamis [9,10]. However, slow onset climate-change-related events such as heat waves and cold spells, while not immediately deadly [11], can compromise farmers’ health and capacity to work.

Heat waves are anticipated to become more common, last longer and have greater intensity [12]. Extreme heat can result in health issues ranging from mild heat stress symptoms, such as headaches and fatigue, to severe heat strokes and fainting [13,14]. Extreme heat can also lead to death during and after heat waves [15,16], and can impair mental capabilities [17]. Heat stress is considered to be a combination of an external thermal environment and the internal heat generated by physical activity [18]. When temperatures exceed more than 98.6 °F (37 °C), sweating is the primary mechanism of cooling down the body, but it is impaired by high air humidity, thereby creating heat-related health problems [19].

While climate change literature, including the Intergovernmental Panel on Climate Change (IPCC) Report 2014, strongly focuses on the increase of warm temperatures, it has remained silent on the health impact of cold spells [20]. Cold temperatures and cold spells are also on the rise in some areas as climate variability increases [21]. Stress on the human body from extreme cold can cause death from hypothermia. According to an international study analysing over 74 million deaths in 384 locations across 13 countries, extreme cold kills 20 times more people than extreme heat [22]. In many countries, the temperature does not reach such extreme lows, and, for the most part, people utilise behavioural thermoregulation in the cold [23]. However, there may be situations where these behaviours are inadequate, such as when impoverished people cannot afford adequate clothing or do not have access to heating. 

As with heat, extreme cold can also negatively affect the health system, through increases in the occurrence of viral flu, cough, cold diarrhoea, asthma, pneumonia, and other respiratory problems [24]. In the cold, vasoconstriction and lowering of tissue temperatures cause numbness, which reduces manual dexterity and strength [19,25]. Extreme cold can also cause cardiovascular diseases, although to a lesser extent than in cases of heat [26], while older, marginalised and underprivileged people are the most affected by extreme cold [27]. The risk of suffering frostbite, for example, increases with age [28]. Unintended cold exposure can also lead to various health hazards and mortality for those people working outdoors, or more impoverished people who cannot afford indoor heating [29,30].

Extreme heat and cold have impacts on workers’ daily activities and work, which require proper coping mechanisms to minimise the impacts of extreme temperatures. Temperatures of 90 °F (32.22 °C) and above or 50 °F (10 °C) and below can detrimentally affect work performance [31]. Exposure to extreme and prolonged heat has led to reduced worker enthusiasm and performance at their work; at the same time, a natural reaction of self-pacing working activities to maintain inner core body temperature will reduce working capacity and lower workers’ productivity [18,32,33,34,35,36,37]. There is an extensive body of literature assessing labour productivity losses from the heat in outdoor and labour intense sectors, such as agriculture [25,32,37,38,39,40,41], mining [18,25,42] and construction [43,44,45]. 

Heat [45,46] and cold [47] can also lead to increased accident rates of outdoor workers. Recent studies have shown that even the urban population is under extreme heat stress and feels impaired in their daily activities and work, particularly in countries where air-conditioning is still under-used [48]. While few studies have investigated the impacts of climate-change-related extreme cold on outdoor workers’ performance and labour productivity [49,50] and their adaptation strategies against cold spells [51], it has been found that extreme cold causes an unpleasant sensation and thermal discomfort. Discomfort may be a distracting factor reducing work performance through the loss of concentration and alertness, and may also cause physical injuries and accidents in the workplace [49].

This study aims to assess the social and economic impacts of climate change using a case study from Nepal. We specifically aimed to (1) assess the impacts of heat waves and cold spells on farmers’ health and levels of heat and cold stress, (2) to explore which factors determine productivity losses, and (3) to reveal the strategies that farmers follow to relieve heat and cold stress and labour productivity loss from extreme temperature.

We used self-reported measures of health and productivity loss, obtained from a survey conducted among 350 farmers in the Terai lowlands in Nepal. This region is considered to be the ‘food bowl’ of the country, and significantly contributes to the national economy. Based on the data source of the Disaster Information Management System (DISINVENTAR) of the United Nations Office for Disaster Risk Reduction (UNISDR), throughout Nepal, 647 cases of cold spells and 49 heat cases of heat waves were reported from 1970 to 2013 [52]. During this time, 822 cold-related and 49 heat-wave-related deaths were recorded. Of these cold-related deaths, 89% percent of deaths took place in the Terai region. The government of Nepal has identified 30 different types of disaster [53]; among these disaster events, the cold spell is considered to be the crucial extreme events that caused the significant damage to agriculture, livestock and human beings. During 1970–2013, economic loss from cold spells was US $835 million, 269,000 Ha of crop land were damaged and 732 cattle were lost due to cold spells [52]. The effect of cold spells has been found to be higher in the low lying Terai region than in the mountain regions, where there is cold in most of the time, but it is not so significant because the population is both sparse and more adapted to the cold climate [54]. On the other hand, the impact of cold is severe in Terai, where the largest share of the population resides, most of them living below the poverty line [54]. Pradhan, Sharma and Pradhan [54] further reported that cold-wave-related deaths increased at the rate of 13% per annum during 1970–2013. So far, there has been only one study from Nepal [55] on how working people in the Terai region respond to heat waves. They concluded that males were found to be highly affected by heat waves, and only a few workers had adapted to using heat wave coping mechanisms.

## 2. Materials and Methods 

### 2.1. Study Area 

The Terai region covers only 14% of the total land area of Nepal, but contributes 72% of the national rice production and 63% of wheat [56]. It is, therefore, referred to as the ‘granary’ of Nepal, with more than 84% of farm households actively engaged in rice production. The region covers 22 districts (out of the 77 districts of Nepal), and is home to more than half of the country’s population of 28.5 million [57]. Based on the Central Bureau of Statistics (CBS) climate change impact survey, 2016, and discussion outcomes with the Nepal Department of Hydrology and Meteorology (DHM) officials, we selected two districts in which to conduct this study, the Banke and Bardiya districts. From these districts, we selected municipalities and their respective wards (lower administrative division) that had been profoundly affected by EWEs in recent years [58]. Over the previous two decades, the Banke and Bardiya districts had recorded, respectively, highest maximum summer temperatures of 118.4 °F (48 °C) and 113 °F (45 °C), and lowest minimum winter temperatures of 32 °F (0 °C) and 39.2 °F (4 °C). 

There is no universal definition of a heat wave in Nepal. In general, a heat wave is locally known as ‘loo’ and prevails during the hot summer months. Based on discussion with district officials, they stated that a ‘loo’ normally occurs in the lowlands when the temperatures reach at least 40 °C and continue for a minimum of two days. Heat waves can cause reductions in crop productivity [59], and the death of livestock and people. From 1974 to 2013, 45 heat-wave-related deaths were reported, solely in the Terai region [54].

A cold spell is a sudden drop in temperature, taking place within 24 hours, and generally is accompanied by thick fog and lasts for many days, a condition known in Nepal as ‘sitlahar’. While cold spells are most common in winter in the low lying Terai region, their occurrence has increased substantially in the lowlands during the last 14 years [60]. Cold spells cause damage to crops [61] and compromise people’s quality of life [60]. Since 1990, cold spells have caused the death of 821 people, primarily in the Terai region (88%; 721 deaths) [61]. Cold spells are currently considered a serious problem affecting Nepal’s food security.

### 2.2. Sampling

**Figure 1:**
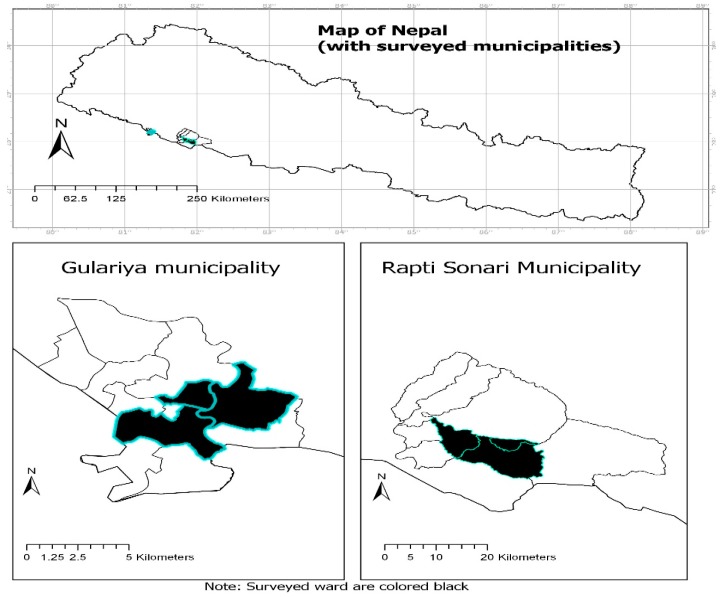
Study areas.

Three wards (5, 8 and 12) of the Gulariya municipality in the Bardiya district and three wards (3, 4 and 5) of the Rapti Sonari rural municipality in the Banke district (shaded areas in Figure 1) were selected purposively. From these purposively selected wards of each municipality, farming households were selected by using systematic random sampling. In total, 350 household heads or main family members were interviewed. Among these sampled households, 52% of interviewed households were from Rapti Sonari and the remaining 48% were from Guleriya. The survey was conducted from the first week of November 2017 to the third week of January 2018 by three experienced and trained research assistants who spoke Nepali, the main language used for the survey, and who could also understand Tharu and local dialects. Ethics approval to conduct this reseach was obtained by the Charles Darwin University Human Research Ethics Committee (H17110).

### 2.3. Questionnaire and Variables

A structured questionnaire was used, which was first pre-tested with 15 respondents randomly selected from villages near to the study area. We asked around 30 questions, which took about 25 minutes, on average. The revised final survey included questions focused on three themes: (i) the farm socio-economic characteristics, (ii) EWEs and their perceived impacts on farmers’ health and labour productivity, and (iii) the existing adaptation strategies used to mitigate the impact of climatic extremes. Farmers were asked to rate whether previous heat waves and cold spells affected their labour productivity, their own health and the health of their families during the previous five years (2012–2017).

Labour productivity loss was defined as a loss in production or not meeting set work targets [35]. Labour productivity loss arises from presenteeism (when at work but unable to perform at full capacity) and absenteeism (not being at work at all) [62]. These concepts have been used extensively to assess labour productivity loss from chronic health issues [63,64]. These studies used self-rated measures of perceived presenteeism and absenteeism with recall periods of 1 month [64], 3 months [65] or a year [34,66]. It was decided to use an extended recall period of five years, in order to capture both good and bad years of extreme heat and cold.

The five year period, furthermore, allowed the study to extend its focus beyond a number of variables common to subsistence farming; the households’ head and other household members often work in the agricultural sector most of the time during cropping seasons, but during non-cropping seasons, they either remain unemployed or are partially involved in other off-farm activities. To cover periods of farm work, the recall period had to be extended to five years. Harvests are very volatile from year to year, and so is the farmers’ workload. Thus, to capture years in which farmers worked full time on their farms, a long recall period was used. A five year or longer recall period has also been used elsewhere in climate change perception studies [60,67,68,69]. 

Perceived stress from heat and cold was measured on a five point scale (‘Never,’ ‘Yes, rarely,’ ‘Sometimes,’ ‘Often’ and ‘Very often’). The associated question was: “*Have you felt that you have been heat (cold) stressed during heat waves (cold spells) when undertaking your agricultural activities in a usual year during the last five years*?” (see Table S1 in Supplementary Materials)

Those respondents who were least stressed by heat and cold were then asked to state their perceived labour productivity. Responses were also measured on a five point scale (‘Never,’ ‘Yes, rarely,’ ‘Sometimes,’ ‘Often’ and ‘Very often’). The related question was: “*If you felt heat (cold) stressed, did you find yourself, as a consequence, less productive when working on agriculture-related activities in the last five years*?” Similarly, farmers were asked open ended questions “*What preventative measures do you currently adopt to avoid heat/cold related stress in the agricultural fields*?” These responses were listed and coded for further analysis. Heat-wave- and cold-spell-related questions had separate sections in the survey instrument, and were asked separately during the households’ survey.

### 2.4. Potential Determinants of Stress and Productivity Loss during Heat Waves and Cold Spells

There has been an increase in studies over the last two decades that explain the factors that lead to human stress from environmental conditions, such as from extreme weather conditions and natural hazards [55,70,71,72]. Previous studies [14,73,74] have found that both physiological and psychological factors influence vulnerability to extreme temperature. The variables included to explain farmers’ heat stress were chosen and categorised, as conducted by Zander, Moss and Garnett [71], and Kovats and Hajat [13] in their studies (Table 1). Potential determinants were sorted into the following four categories: socio-economic (land size, income, access to various facilities, type of housing, and livestock); psychological (perception and experience of extreme heat waves and cold spells, and level of work satisfaction in agriculture); physical (age, number of active household members, gender and health status, average working days in agriculture during summer and winter seasons separately, and implemented heat wave and cold spell adaptation measures); and environmental factors (respondents’ location in urban or rural areas).

It was assumed that the same factors that affect cold and heat stress also affect associated productivity loss. Studies on extreme heat have shown that there is a strong correlation between the two [71,72]. The impacts of cold spells are associated with several factors, consisting of individual, socio-economic, climatic, clothing availability, and physical activity [49]. As the literature on cold stress is sparse, it was assumed that most factors that affect heat stress also affect cold stress.

#### 2.4.1. Social Factors

People with higher income and wealth (farm size, self-reported annual income) are less likely to be heat stressed [13,73,75,76], probably because wealthy people have a higher adaptive capacity to cope with extreme heat. Similarly, those farmers who have access to actual weather information are assumed to be more likely to perceive extreme events and take household level adaption measures in response [77]. They would, therefore, be less likely to suffer from heat and cold stress and less likely to observe labour productivity loss from heat and cold.

Those residing in concrete or well-built houses are less likely to report heat and cold stress. It could be that extreme-temperature-resilient houses allow for a sound sleep during the night, and thus workers may be less stressed while working in the field during hot days [78]. Workers with well-built and comfortable houses are, therefore, assumed to be less stressed and vulnerable to extreme temperatures, and less likely to report productivity loss than workers with poor housing [55,79]. Farmers in better houses are also more likely to have better quality sleep during extreme temperatures, which could increase their working capacity in the following day [34]. 

#### 2.4.2. Psychological Factors

It is assumed that people who have had past experience with climate extremes are more likely to be worried and stressed by them [80,81]. Direct experiences of past climatic events will have a substantial impact on risk perception [82,83], and may encourage farmers to take precautionary adaptation measures.

As job satisfaction is one of the crucial determinants of labour productivity improvement, the study further assumed that workers who were satisfied with their jobs were highly productive [84,85]. An individual with a poor existing health condition leads to considerable labour productivity loss while performing a physically demanding job compared to a healthy worker [86,87].

#### 2.4.3. Physical Factors

Older people and those with illnesses are particularly vulnerable to the impacts of both heat and cold [88], and heat alone [14,89]. Thus, it was expected that households with more active family members of working age, between 15 and 59 years old, would be less likely to suffer from heat and cold stress, as they could help each other to perform agricultural activities during extreme weather conditions and be less exposed to the extreme weather. Those farmers who had a higher number of family labourers on their farms were expected to be less stressed and less compromised in their productivity [32]. In addition, those who work physically hard outside, in industries including the agricultural, construction, mining, and military sectors, are also vulnerable to the impacts of weather extremes [90]. 

Workload and work intensity during extreme temperatures (length of exposures to extreme temperature or task duration) is expected to have a positive impact on the levels of heat and cold stress, and a negative impact on labour productivity [18,31,44,50,55]. Reduced labour productivity is a function of environmental humidity, radiant heat, air movement and ambient temperature [19]. Work performance is a function of physical, mental, social and psychological factors [91], because heat waves can have negative impacts on workers’ productivity due to thermal stress on human cognitive and physical factors [92]. Farmers who usually work in the fields under high temperatures have been found to be affected by a range of heat-related health problems, such as exhaustion, irritability, sleeplessness or having difficulties in maintaining work level and output [41].

Health is one of the dominant factors affecting susceptibility to both heat stress [35,71,72,93,94] and cold stress [74,95]. Employees with recurring illness and painful symptoms are more likely to report heat and cold stress than employees without these health problems [74,79,95]. Similarly, older people are highly susceptible to impacts of extreme heat [71,96,97], because elderly people are more often physically inactive and of poor health. It was additionally expected that farmers who had already adopted numerous climate change-related adaptation measures would be less worried and stressed about future extreme weather because they might think that they are sufficiently prepared for it. The nature and effectiveness of climate change responses could play a crucial role in further implementing risk mitigation behaviour [98,99].

Men are assumed to be more stressed by heat than women [55,76], as men are exceedingly exposed to heat in physically demanding outdoor activities (farming, mining and construction work). Other studies have reported that men and women have slightly different physiology, endocrinal physiology and body characteristics, specifically that women have a larger surface to mass ratio, which implies that women are more prone to heat loss [18].

The availability of weather information leading to greater awareness, and participation in community organisations or other social networks are expected to influence farmers’ behaviour in response to climate-change-related extreme events [68,77].

The level of physical exertion is a strong predictor for heat stress [34,44,71]. This study assumed that farmers who own livestock conduct more labour-intensive tasks that are required to be performed during extremely hot and cold days, such as fetching fodder. This study, therefore, assumed a positive relationship between owning livestock and heat and cold stress. As for labour productivity loss, there was also assumed to be a higher overall loss, as animals are also affected by extreme heat and cold [100,101].

On the other hand, farmers with livestock such as buffalo can rely on their aid for some labour-intensive activities, including the pulling of carts to carry agricultural products and agricultural inputs from and to the agricultural field and markets, in which case they might be less effective in their productivity during extreme temperature. We also expected a positive relationship between farmland size and labour productivity loss because of the expected higher workload with more land. 

#### 2.4.4. Environmental Factors

Urban residents were found to be more heat stressed [13,93,102] because the phenomenon of the urban heat island aggravates heat stress. The study also assumed that urban residents are less likely to be stressed due to cold spells because of the urban heat island problem.

### 2.5. Data Analysis

Ordered logit regression models were estimated to examine the impact of various controlled variables on the farmers’ level of heat stress and cold stress during 2012 and 2017. The initial responses on a five point scale were reduced to three points. The first two heat and cold stress levels (‘Never’ and ‘Rarely’) were grouped into the first point, while the third and fourth levels (‘Sometimes’ and ‘Often’) were grouped into the second point. The third point included only the highest heat stress level, ‘Very often’ (Section 2.3). The dependent variable, therefore, took on the values 1 to 3, ordered from low to very high stress levels.

For the assessment of productivity loss, binary logit regression models were run. The dependent variables were perceived as labour productivity loss from heat stress (cold stress), coded 0/1. 

Responses were initially separated into four categories for both the heat wave and cold spell models. The levels of responses were, therefore, grouped according to productivity loss: “Definitely not” and “Probably not” were assigned to 0 (“Not perceiving labour productivity loss”), while 1 (“Perceived labour productivity loss”) included “Definitely Yes” and “Probably yes”. Separately ordered logit and binary logit analyses were also estimated to examine the effects of various explanatory variables on the levels of heat and cold stress and self-reported labour productivity loss from extreme temperatures at the district level respectively. 

**Figure 2:**
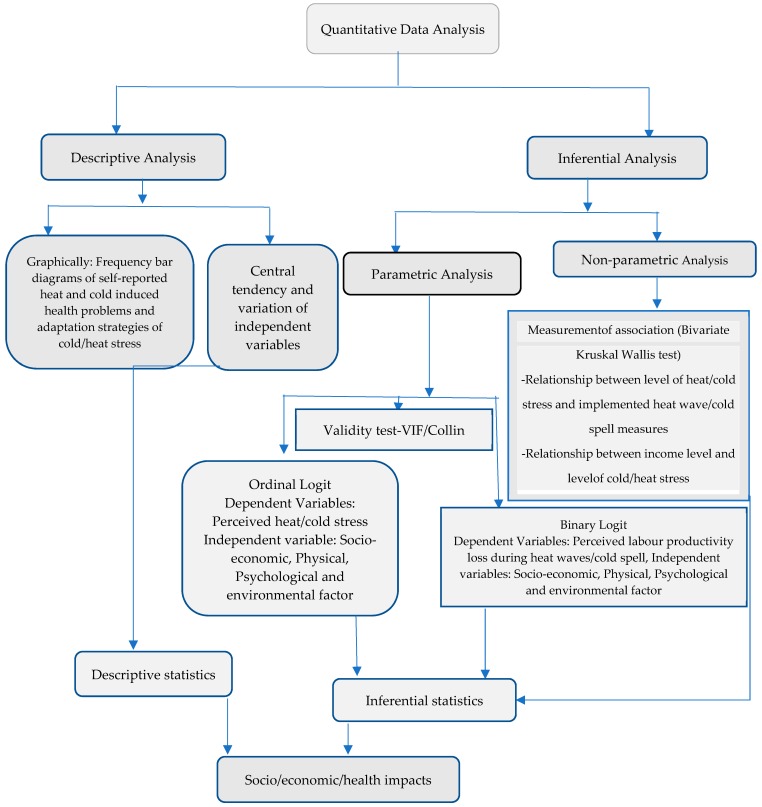
Analytical framework.

A bivariate relationship was analysed using a Kruskal–Wallis test to examine the relationship between heat and cold-related responses and other explanatory variables, such as the level of household income and the level of heat and cold stress. Multicollinearity was tested for using the ‘Collin’ command in STATA. The mean variance inflation factor (VIF) was less than 10 (Mean VIF < 1.89), meaning that there was no indication of correlation [103]. The correlation among the included explanatory variables did not exceed 0.56, thus no correlation exists, as shown by correlation matrices (Tables S4–S7 in Supplementary Materials). For details, see the following analytical framework (Figure 2).

## 3. Results

### 3.1. Sample Description

The average age of the respondents was 38.7 years (SD: 13). Approximately 62% were male, and ~67% had some formal education (Table 2). The average household size was 7.8 persons (SD: 5.31), and farmers’ average experience in the agricultural sector was 21.2 years (SD: 12.6). Among the total respondents, nearly 38% were female, and nearly one third of the total respondents never attended school, while ~32% had completed high school.

The mean household monthly expenditure was NPR 16,130 (USD = NPR 107.10, source: https://www.nrb.org.np/fxmexchangerate.php, 8 June 2017) (SD: 18000), which was less than the national monthly household expenditure of NPR 25,928 in 2016 [104]. Income was equally distributed among the categories (Table 2).

Nearly 33% of households reported that they had access to actual weather information. The average land holding was 1.42 Bigga (1 Bigga = 0.6772 ha), and 75% of respondents owned their land. About 53% of farmers perceived their health as good and only 4% as poor. Approximately 16% of respondents reported that they were a little stressed during heat waves (‘low levels’), ~38% moderately, and ~47% severely stressed. Similarly, approximately 15% were a little cold stressed, ~45% moderately cold stressed, and ~ 41% were severely cold stressed. When comparing the means of various independent variables across the two study districts by using t-tests (Table 2), significant mean differences were observed in all the variables except cold spell perception, level of perceived heat stress and satisfaction with existing health status. 

### 3.2. Heat- and Cold-Related Illnesses and Injuries

Thirty seven per cent of respondents had experienced heat-related health problems and 34% cold-related problems in the last five years, from 2012 to 2017. Respondents made distinctions of diseases and symptoms based on winter and summer seasons. Nearly half of respondents thought that their health condition had been negatively affected during heat waves (48%) and cold spells (51%). About 8% of respondents had been highly affected by both cold spells and heat waves. Only 4% and 3% of respondents, respectively, reported without a doubt that their health had not been impacted by heat and cold.

**Figure 3:**
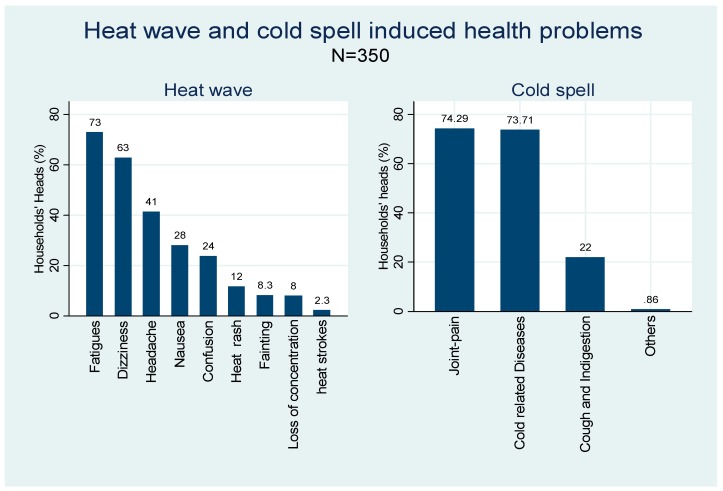
Heat wave and cold spell induced health problems.

Those farmers’ who experienced extreme heat- and cold-related illnesses were further asked about their experience. On average, farmers reported three heat-related and two cold-related illnesses. The most commonly reported illness relating to heat was fatigue (73%), followed by dizziness (63%), headaches (41%), nausea (28%), confusion (24%), heat rashes (12%), fainting (8%), loss of concentration (8%) and heat strokes (2%). Joint pains (74%), pneumonia and respiratory problems (74%), and cough and indigestion (22%) were the main illnesses relating to extreme cold (Figure 3).

### 3.3. Determinants of Farmers Perceived Heat and Cold Stress

The results from the ordered logit model showed that farmers with access to actual weather information were less likely to report heat (*p* < 0.01) and cold (*p* < 0.01) stress than those without this information (Table 3). Owning livestock had a significant positive impact on perceived heat (*p* < 0.1) and cold stress (*p* < 0.05). Respondents who perceived an increament in the frequencies of heat waves and cold spells were more likely to have reported higher heat (*p* < 0.01) and cold stress levels (*p* < 0.01). Farmers who had implemented more heat wave and cold spell adaptation measures in the past were more heat (*p* < 0.01) and cold stressed (*p* < 0.01).

Age (*p* < 0.05) and health (*p* < 0.01) had significant positive impacts on the perceived levels of heat, but not cold, stress. Farmers from urban areas reported higher cold stress levels (*p* < 0.01) than those from rural areas, while farmers who worked more days outdoors in agricultural activities during the summer season reported higher heat stress (*p* < 0.1). District level analysis of determinants of farmers perceived levels of heat and cold stress also presented in the supplementary Table S2. 

### 3.4. Labour Productivity Loss during Heat Waves and Cold Spells

Farmers’ perceived heat and cold stress levels, and the number of associated illnesses or symptoms, to significantly increase labour productivity loss during heat waves (*p* < 0.05) and cold spells (*p* < 0.05) (Table 4). Farmers in urban areas were more likely to report productivity losses during heat waves (*p* < 0.01) and cold spells (*p* < 0.01) than farmers in rural areas. Respondents who had access to actual weather information were more likely to perceive labour productivity loss from heat waves (*p* < 0.01) and cold spells (*p* < 0.01) than those without this information. Respondents who had implemented more heat wave (*p* < 0.01) and cold spell (*p* < 0.1) adaptation measures (such as clothing adjustment, rescheduling working times, rest breaks) in the past were more likely to perceive labour productivity loss during heat waves and cold spells.

More variables affected farmers’ productivity loss during cold spells than during heat waves. Respondents with higher annual income (*p* < 0.05) were more likely to report labour productivity loss during cold spells than those with lower income. Male respondents were less likely to perceive labour productivity loss from cold spells than female respondents. Age was significant (*p* < 0.05) and positive, but negative when squared (*p* < 0.05), which indicates that reported labour productivity loss increased with age but decreased eventually. District level analyses of self-reported labour productivity loss from extreme temperature are shown in the supplementary Table S3. 

About 31% (32%) of household heads stated they had been absent from field work during cold spells (heat waves). Those who reported absenteeism during heat waves, had, on average, missed 16 days of farm work during the past year. The average number of absent days during cold spells was 11.5 during the past year. At the same time, about 85% of respondents reported that more than 50% of their work time was less productive during heat waves, and 64% of respondents reported that more than half of their working hours were less productive during cold spells. It could be that cold spells normally occur during the winter season when agricultural activities are limited. 

### 3.5. Adaptation and Relief Strategies to Cope with Heat and Cold Stress

Nearly 96% of respondents said that they wear broad-brimmed hats or used umbrellas to protect themselves from extreme heat when working on the farm. Some 93% of respondents who were heat stressed reported heat relief measures, such as resting in the shade and slowing down their working pace, while ~61% stopped their outdoor farm activities during extreme heat waves. Nearly 65% of respondents rescheduled their working shifts to moderate the impact risks of heat on their health and labour productivity. Approximately 17% of respondents adopted cooling techniques when working outside on very hot days, while ~54% stated that they had different means to cool down, such as drinking more cold water, staying in sheds, staying inside the house, and using wet clothing to reduce the impacts of heat.

Of those respondents (285) who wanted to shift their working schedules, 17% preferred to start and finish earlier, and only about 1% preferred to start and finish later. About 82% wanted to work early in the morning and late in the evening on very hot days to avoid the hottest hours. About 12% did not change their working plans at all, 22% changed their plans rarely, 61% changed sometimes, and 6% often or very often. About 42% of the respondents regularly hired additional labourers to get the work done during hot days, and further reported that nearly all those respondents found their labourers to be less productive during very hot days.

Similarly, to avoid and mitigate the impacts of extreme cold, farmers used the following adaptation measures: wearing warm clothes (99%), cessation of work if the temperature dropped or resting to warm up (82%), rescheduling working timetables (82%), and drinking hot beverages (65%). Of those who rescheduled their working times, most (94%) preferred to work in the daytime during very cold days. Stopping work (χ^2^ (2) = 5.035, *p* = 0.0807) and rescheduling working time (χ^2^ (2) = 10.39, *p* = 0.0055) were the two heat-related responses most affected by the level of heat stress farmers experienced. 

Less stressed farmers were less likely to stop working, or to reschedule their working schedules, than highly stressed farmers. Stopping work and resting to warm (χ^2^ (2) = 30.56, *p* = 0.0001), rescheduling working hours (χ^2^ (2) = 7.556, *p* = 0.0229), and drinking hot beverages (χ^2^ (2) = 75.35, *p* = 0.0001) were most highly affected by the level of cold stress. All the heat- and cold-related response strategies were more significantly affected by income level (Table S8 in the Supplementary materials).

## 4. Discussion

### 4.1. Health Impact of Heat Waves and Cold Spells on the Farming Community

We found that health status has a significant impact on farmers’ perceived heat stress, but not on cold stress. Rocklöv et al. [105] stated that health effects from heat waves would appear within 1–2 days and are relatively easy to identify. Health effects from cold spells, however, are more likely to be associated with higher mortality and appear within two weeks following exposure, and it is difficult to infer causality between health effects and cold spells. This is supported by a previous study [61], which found that mortality risks associated with cold spells (721) had, since 1990, been reported as being almost 16 times higher than heat wave reported deaths (45) since 1978 in the study region.

The most common heat wave related health problems among Nepalese farmers are fatigue, dizziness and headaches, followed by nausea, fainting, confusion and heat rashes. These results confirm findings in other studies from Nepal [55], India [14,35] and other parts of the world [13,72,106]. The most common symptoms during cold spells are joint pain (arthritis), cold-related diseases (such as respiratory problem, pneumonia, cold cough) and indigestion problems, as was also reported by Hassi, Rytkönen, Kotaniemi and Rintamäki [74], and Davídkovová et al. [107].

People who already suffer from health problems such as cardiovascular diseases [74,108], pre-existing diabetes (indigestion) and respiratory diseases [96], and musculoskeletal disorders [49,51] are usually more vulnerable to the effects of heat waves and cold spells. Musculoskeletal disorders are considered to be a significant hazard of agricultural occupations, and can cause labour productivity loss and even disability [108]. As these illnesses are related to age [15,71,97,105,106,109,110], it was not surprising that older farmers reported higher levels of heat stress.

### 4.2. Determinants of Self-Reported Heat and Cold Stress

The land size variable did not have any significant impact on the levels of heat and cold stress. Farmers who stated themselves to have regular access to information on actual weather phenomena were less likely to perceive future heat and cold stress. Respondents were less worried and stressed about upcoming weather conditions because they were well informed about potential coping mechanisms in advance, and thus more likely to implement relevant adaptation strategies [111], which could reduce the levels of heat and cold stress. Some demographic variables were also not significant, including education and income. It was expected that better educated and more prosperous farmers would be less likely to be stressed by heat and cold than those with lower education and income, a result found elsewhere [73], because they might be more aware of heat- and cold-related coping strategies. Owning livestock had a mixed impact. Respondents who owned livestock were found to be more heat and cold stressed, probably because of the increased need to spend a significant amount of time outside and doing labour intensive tasks related to livestock rearing, such as feeding and providing water, which is even more important during very hot and cold days. Contrarily, owning livestock had no impact on productivity loss during heat waves and cold spells. This might be because the increased labour needed to rear livestock and the expected higher labour productivity loss that might occur during extreme temperatures is offset by the benefits livestock provide as, for example, draft animals. 

As expected, older people reported being highly heat stressed. This is most likely related to older people’s deterioting health [14,89]. Despite adopting various heat wave and cold spell coping measures, farmers were found to suffer additional heat and cold stress. Potentially, those adopted measures were not very effective in reducing the weather risks caused by extreme events [98,99] in the study area, such as heat waves and cold spells. It was expected that variables associated with a higher workload and intensity (working more days) had positive impacts on the level of heat and cold stress, but this study found a positive impact only in the context of heat stress, which is consistent with the findings of previous studies [18,55,112].

As assumed, past experience with extreme weather events such as heat waves and cold spells was positively associated with the levels of heat and cold stress [80,82]

Urban respondents suffered more cold stress, which could be because the majority of farmers in the urban areas were poor and their housing conditions were not cold resilient. Due to long term cold exposure resulting from factors such as poor housing conditions, the risk of hypertension due to cold stress may be increased for outdoor workers, such as farmers [49].

### 4.3. Impact of Heat Waves and Cold Spells on Labour Productivity

Respondents who reported higher levels of annual income were more likely to perceive labour productivity loss from cold spells. This was surprising, because higher incomes usually provide better opportunities to implement coping mechanisms [113], but a lack of awareness for cold spell protection mechanisms, and farmers’ decreased motivation to work could potentially explain the perceived labour productivity loss during cold spells. 

Farmers with access to weather information were found to be more likely to perceive labour productivity losses from heat waves and cold spells from 2012 to 2017. This was also surprising, as information about the weather would have aided them in preparing to take precautionary measures, such as drinking enough cool or hot water and wearing appropriate clothing. They would also have had the opportunity to schedule and plan their work, while taking predicted hot or cold periods into account. However, the quality and accuracy of the weather information to which farmers have access are unknown, and probably not very reliable, as farmers did not take much notice of it.

Age had no impact on perceived productivity loss during heat waves, in contrast with other studies, which have found that age, heat stress, and productivity loss from heat stress are positively correlated [96,97]. In the cold spell model, however, age did have the expected inverse U-shape relationship with productivity loss. Those farmers of increased age self-reported higher cold stress levels, as they were more active in their physical work [88]. Level of cold stress declined after a certain age when they were less involved in physical outdoor activities. 

Men were less likely to perceive labour productivity loss from cold spells than women. The peripheral vasoconstriction of women inhibits their ability to maintain safe skin temperature in extreme cold, as they have less maximum heat production capability and lower mean foot, hand, and skin temperatures, and have a relatively higher risk for cold injuries [113]. In order to maintain their body temperature, women require better clothing insulation, which increases hobbling effects and hinders dexterity [113,114].

Respondents who had experienced many heat and cold-related illnesses from 2012 to 2017 were more likely to perceive labour productivity loss from heat waves and cold spells. This might be linked to their health status, meaning those who experienced many heat- and cold-related illnesses were unhealthy, and therefore more prone to stress than healthy respondents [71], which in turn hampered their working capacity. Likewise, farmers who reported respiratory symptoms and pulmonary obstructions, as triggered in cold weather, were more likely to be less productive during cold spells [74,112]. Farmers implemented a number of different coping strategies in response to EWEs, but their perceived labour productivity loss from heat waves and cold spells remained high. The number of adaptation mechanisms implemented might not have been sufficient enough to reduce the negative impacts of EWEs. Another reason for perceived productivity loss could be the increasing magnitude and frequencies of extreme temperatures in recent years. 

As expected, farmers who perceived a moderate and high level of heat and cold stress were more likely to report labour productivity loss than farmers who perceived lower levels of heat and cold stress [34].

We found that urban farmers (Bardiya) were more likely to perceive labour productivity loss due to both heat and cold. For heat, at least, this result was not surprising, as the urban population is highly affected by temperatures increasing due to the urban heat island effect [93]. As this study was conducted in the warm and humid region of Nepal, the effects of cold spells on health and labour productivity loss were probably higher than in other regions of Nepal, per farmer acclimatisation levels. Heat effects are generally lower in areas with higher long-term temperatures, because people have adapted to the higher average temperatures [108]. As expected, therefore, cold effects were found to be higher in communities with warm temperatures. 

### 4.4. Adaptation Measures Against Heat Waves and Cold Spells

Farmers adopted multiple strategies simultaneously. The most applied precautionary measure for protection against direct heat exposure while working in agricultural fields was the use of hats and umbrellas. During extreme heat, farmers would sometime stop their work completely, and preferred to reschedule shifts to minimise heat exposure. Working during the cooler parts of the day, such as early in the morning or late in the evening, is a practice widely found across the low-lying regions of Nepal. Likewise, taking frequent breaks, resting in the shade, and slowing working pace, as has been found in Australia [25], were the other primary heat exposure minimising mechanisms that respondents adopted while working in agriculture. Regularly resting and slowing working pace is a type of behaviour acclimatisation, which helps to reduce bodily heat strain while working in agriculture [115]. 

Similarly, farmers managed various cooling techniques such as bathing in cold water, wearing wet clothes, and drinking a lot of cold water to avoid dehydration from heat exposure, as also found by Pradhan, Sharma and Pradhan [54]. Stopping work and rescheduling work shifts are the two heat response measures found to significantly differ across the three perceived heat stress levels, low, medium, and high. As climate variability increases and temperatures get more extreme, these readily accessible measures are more likely to be abandoned and more expensive (financially, socially and personally) technologies might be needed.

Most of the respondents wore warm clothes to keep them safe from cold spells during winter, thus helping to maintain core body temperature and to protect from adverse health impacts [74]. As most of the farmers in the study areas were impoverished, they were highly affected by decreased temperatures during winter. The local governments in the study areas often issued directives to the people to stay inside, and also provided warm clothes and wood to deprived households during cold spells [54]. Stopping work during extreme cold and altering work schedules were widely practiced coping strategies.

### 4.5. Limitation of the Study

There are two limitations to the study. The first limitation relates to how self-perceived labour productivity loss was measured and the chosen recall period. Quantifying actual labour productivity loss measured in term of absenteeism and presenteeism in agricultural farming households is a challenging task because most of the farming household members are self-employed within the agriculture sector. Farming households are mostly busy during the planting and harvesting times of the cropping seasons, but remain partially or fully unemployed during the off-farm seasons. In that context, calculating the monetary measurement of productivity loss is hard in an unorganised agriculture sector, where there is a lot of seasonal and disguised unemployment. Rather than directly measuring the monetary value of labour productivity loss, we instead measured self-perceived labour productivity loss in the ordinal scale, while farmers were involved in the agriculture sector between 2012 and 2017. We chose five years as the recall period. The method might suffer from recall bias, because respondents may not accurately and precisely remember previous events or their experience after such a long time, or their memories might have been distorted by other experiences and events [116]. To minimise the recall bias, we carefully designed the research questions and implemented appropriate research tools. Rather than assessing the perceived productivity loss as an exact number or percentage, we allowed respondents to answer on an ordinal scale. The second limitation of the study relates to the fact that the study used cross sectional data. Since the data were collected at a single point of time, we were not able to determine the actual cause and effect relationship [117] between the proposed perceived heat and cold stress and perceived labour productivity loss and actual extreme temperatures. Thus, future studies should be conducted over a series of data collection waves, producing longitudinal data that can allow for climatic conditions and the occurances of EWEs across the study regions. Additionally, the results of our study would further benefit from information from local and regional hospitals on the number of patients admitted and discharged during heat waves and cold spells. 

## 5. Conclusions

This study found that individual farmers and their family members had experienced various heat-wave- and cold-spell-related illnesses and health problems between 2012 and 2017. Fatigue, dizziness, headaches, nausea, confusion, heat rashes, fainting, loss of concentration and heat strokes were the most common health problems self-reported by farming households during heat waves. Likewise, joint pain, pneumonia, respiratory problems, cold cough and indigestion were the common health issues that farmers were mostly suffered during cold spells. Though farming households had been highly affected by both forms of EWE, heat waves and cold spells, in recent years, the impact of cold spells was found to be higher on farming households. Potential reasons for this could be that there was higher acclimatisation to heat waves, and less adaptation towards cold spells, due to a limited coping capacity caused by relative poverty and farmer ignorance. Farmers were found to apply broad-brimmed hats or umbrellas, resting in the shade, slowing down their working pace, and completely stopping work during extremely hot days, rescheduling their working schedules, and applying various cooling techniques to reduce the impact of heat stress on labour productivity loss from heat waves. The main coping mechanisms used as precautionary measures to mitigate labour productivity loss during cold stress included wearing warm clothes, stopping work, resting to warm up, rescheduling working timetable, and drinking hot beverages. To help mitigate the effects of extreme weather events and save lives, public awareness campaigns should specifically target the susceptible parts of the population with information on the appropriate actions to take during extreme temperatures. Extreme temperature warnings based on weather forecasts should also be publicly broadcast, as well as heat and cold stress prevention measures. The implementation of risk communication and risk awareness through local media, providing information about the possible consequences of heat waves and cold spells, and the potential coping mechanisms, could be a primary strategy by which to mitigate potential health impacts and labour productivity losses.

## Figures and Tables

**Table 1:** Summary of factors determining the heat and cold stress and related productivity loss.

Factor	Impact	Source
**Social factors**		
Income	Negative	Kovats and Hajat 2008, Tawatsupa et al. 2010, Gronlund 2014, Zheng and Dallimer 2016
Access to weather information	Positive	Bryan et al. 2009, Bryan et al. 2013
Type of house	Positive	Gifford and Zong 2017, Zander et al. 2015, Pradhan et al. 2013
Education	Positive/Negative	Gronlund 2014
Livestock	Positive/Negative	
**Psychological factors**		
Experiences of heat waves and cold spells	positive	Venugopal et al. 2015, Akerlof et al. 2010, Akompab et al. 2013, Wachinger et al. 2013
Satisfaction with job/work	Positive	Kramer and Hafner 1989, Baruch-Feldman et al. 2002
Existing health condition	positive	Dollard and Neser 2013, Burton et al. 1999
**Physical factors**		
Age	Positive	Hansen et al. 2011, Sun et al. 2016, Zander et al. 2017, Hajat et al. 2014
Male	Positive/Negative	Tawatsupa et al. 2010, Pradhan et al. 2013, Burse 1979, Lundgren et al. 2013b
Current health status/pre-existing extreme-temperature-related symptoms/illnesses (numbers)	Positive	Hassi et al. 2005, Rocklöv and Forsberg 2008, Gifford and Zong 2017, Mathee et al. 2010, Zander et al. 2018a, Burton et al. 1999
Implemented response measures	Positive	Zaalberg et al. 2009, Wise et al. 2014
Length of exposure to extreme heat/cold	Positive	Lundgren et al. 2013, Pilcher et al. 2002, Acharya et al. 2018, Enander 1987
**Environmental factors**		
District/urban/heat island effects	Positive	Kovats and Hajat 2008, Kleerekoper et al. 2012, Zander et al. 2018a

**Table 2:** Sample description (N = 350).

Variables	Bardiya Frequency (%)	Banke Frequency (%)	P-Value	Overall Sample Frequency (%)
***Sample Households***	167 (47.71)	183 (52.29)		350 (100)
***Socio-economic***				
Land size (Bigga) (mean; SD)	1.22 (1.47)	1.63 (1.94)	0.02	(1.42; 1.81)
Annual household’s income (NRP)			0.001	
<50000	12 (7.1)	24 (13.1)		36 (10.2)
50,000–100,000	38 (22.7)	41 (22.4)		79 (22.5)
100,000–200,000	31 (18.5)	52 (28.4)		83 (23.7)
200,000–300,000	35 (20.9)	41 (22.4)		76 (21.7)
>300,000	51 (30.5)	25 (13.6)		76 (21.6)
Education			0.02	
No formal education	47 (28.1)	67 (36.6)		114 (32.5)
Primary	58 (34.7)	67 (36.6)		125 (35.7)
High school	29 (17.3)	23 (12.5)		52 (14.8)
Completed 10 + 2	14 (8.3)	16 (8.7)		30 (8.5)
Undergraduate and above	19 (11.3)	10 (5.4)		29 (8.5)
Access to weather information			0.02	
Yes	65 (38.9)	50 (27.3)		115 (32.8)
No	102 (61.1)	133 (72.6)		235 (67.2)
House type			0.0007	
1, If concrete and brick house	71 (42.5)	111 (60.7)		182 (52)
0, Otherwise (leaves, mud)	96 (57.5)	72 (39.3)		168 (48)
Livestock			0.01	
1, If have cows/buffalos	125 (74.8)	115 (62.8)		240 (68.5)
0, Otherwise	42 (25.1)	68 (37.2)		110 (31.5)
***Physical***				
Age (mean; SD)	37.1 (13.3)	40.1 (12.4)	0.03	(38.72; 12.9)
Sex			0.009	
Male	93 (55.6)	127 (69.4)		220 (62.8)
Female	74 (44.3)	56 (30.6)		130 (37.2)
Household size (mean; SD)	7.22 (4.87)	8.49 (5.59)	0.02	(7.82; 5.29)
Health Satisfaction			0.7215	
Not at all satisfied	4 (2.4)	0 (0)		4 (1.1)
Not very	12 (7.1)	17 (9.2)		29 (8.2)
Moderately satisfied	95 (56.8)	107 (58.4)		202 (57.7)
Fairly satisfied	56 (33.5)	57 (31.1)		113 (32.2)
Very satisfied	0 (0)	2 (1.09)		2 (0.5)
Agricultural job satisfaction			0.009	
Not at all satisfied	2 (1.2)	3 (1.6)		5 (1.4)
Not very	23 (13.7)	25 (13.6)		48 (13.7)
Moderately satisfied	133 (79.6)	115 (62.8)		248 (70.8)
Fairly satisfied	9 (5.3)	39 (21.3)		48 (13.7)
Very satisfied	0 (0)	1 (0.5)		1 (0.2)
Perceived health condition			0.02	
Bad	3 (1.8)	11 (6)		14 (4)
Fair	66 (39.5)	83 (45.3)		149 (42.5)
Good	98 (58.6)	89 (48.6)		187 (53.5)
Heat wave measures (mean; SD)	3.8 (0.71)	3.1 (1.08)	0.00	(3.5;0.9)
Cold spell measures (mean; SD)	3.4 (0.87)	3.1 (1.03)	0.00	(3.2;0.9)
Working days in summer (mean; SD)	42.8 (23.4)	47.4 (23.6)	0.06	(45.2;23.6)
Working days in winter (mean; SD)	32.9 (26.08)	43.3 (26.8)	0.00	(38.3;26.9)
Heat-related illnesses over the previous five years (numbers) (mean; SD)	3.13 (1.39)	2.18 (1.44)	0.00	(2.64; 1.49)
Cold-related illnesses over the previous five years (numbers) (mean; SD)	2.04 (0.83)	1.39 (0.88)	0.00	(1.70; 0.92)
***Psychological***				
Level of perceived heat stress			0.209	
Low	23 (13.7)	31 (16.9)		54 (15.4)
Medium	60 (35.9)	72 (39.3)		132 (37.7)
High	84 (50.3)	80 (43.7)		164 (46.8)
Level of perceived cold stress			0.00	
Low	17 (10.1)	34 (18.5)		51 (14.5)
Medium	63 (37.7)	94 (51.3)		157 (44.8)
High	87 (52.1)	55 (30.05)		142 (40.5)
Heat wave perception			0.004	
Increased	156 (93.4)	155 (84.7)		311 (88.8)
Constant	6 (3.5)	9 (4.9)		15 (4.2)
Decreased	5 (2.9)	19 (10.3)		24 (6.8)
Cold spell perception			0.35	
Increased	87 (52.1)	101 (55.1)		188 (53.7)
Constant	22 (13.1)	29 (15.8)		51 (14.5)
Decreased	58 (34.7)	53 (28.9)		111 (31.7)

**Table 3:** Results of ordered logit model with the dependent variables being the level of heat stress and cold stress (from 1 very low to 3 very high).

Variables	Perceived Heat Stress Category	Perceived Cold Stress Category
***Socio-economic***		
Land size (in Bigha ^1^)	−0.03 (0.08)	0.0002 (0.08)
Annual income (1–5)	0.10 (0.10)	0.08 (0.11)
Having access to weather information	−1.03 *** (0.25)	−0.74 *** (0.28)
Living in concrete or brick building	−0.10 (0.23)	0.20 (0.23)
Owning livestock	0.44 * (0.24)	0.48 ** (0.24)
Level of education (1 to 5)	0.11 (0.11)	0.14 (0.12)
***Physical***		
Age	0.11 ** (0.05)	0.05 (0.05)
Age Square	−0.00 (0.00)	−0.00 (0.00)
Number of active family members (15–59 years)	0.02 (0.04)	−0.02 (0.04)
Male	−0.12 (0.26)	−0.01 (0.26)
Health status (1 to 3)	−0.25 (0.21)	0.18 (0.21)
Number of implemented response measures	0.35 *** (0.13)	0.58 *** (0.15)
Number of working days	0.01 * (0.01)	0.001 (0.01)
***Psychological***		
Perceived extreme events experiences (1 to 3)	0.71 *** (0.20)	0.38 *** (0.15)
Health satisfaction (1 to 5)	0.34 ** (0.17)	0.27 (0.18)
***Environmental***		
Living in an urban area	0.07 (0.24)	0.82 *** (0.24)
Constant cut 1	3.81 ** (1.51)	3.53 ** (1.58)
Constant cut 2	5.93 *** (1.53)	6.10 *** (1.61)
Observations	350	350

*** *p* < 0.01, ** *p* < 0.05, * *p* < 0.1; Standard errors in parentheses, ^1^ I Bigha = 0.67 ha. Note: the number of implemented response measures were either in response to heat waves or cold spells, and the number of working days was either during the summer or winter in the heat wave and cold spell model, respectively. The number of perceived events were in relation to either heat waves or cold spells, depending on the model.

**Table 4:** Determinants of self-reported labour productivity loss.

Variables	Perceived Labour Productivity Loss during Heat Waves	Perceived Labour Productivity Loss during Cold Spells
***Socio-economic***		
Land size (in Bigha)	−0.14 (0.14)	−0.05 (0.13)
Annual income (1 to 5)	0.28 (0.19)	0.39 ** (0.18)
Access to weather information	2.22 *** (0.64)	2.60 *** (0.64)
Living in concrete or brick building	0.40 (0.43)	0.41 (0.39)
Owning livestock	0.44 (0.43)	0.03 (0.40)
Education (1 to 5)	0.16 (0.21)	0.23 (0.21)
***Physical***		
Age	0.09 (0.10)	0.22 *** (0.08)
Age Square	−0.009 (0.00)	−0.002 *** (0.00)
Active family members (15–59 years)	−0.02 (0.06)	−0.04 (0.06)
Male	−0.68 (0.48)	−0.75 * (0.44)
Health status (1 to 3)	−0.31 (0.36)	0.21 (0.34)
Number of perceived illnesses/symptoms	0.37 ** (0.15)	0.50 ** (0.23)
Number of implemented response measures	0.88 *** (0.23)	0.43 * (0.24)
Number of working days	0.01 (0.01)	−0.001 (0.01)
***Psychological***		
Perceived extreme events experience (1 to 3)	0.32 (0.34)	−0.02 (0.24)
Perceived stress medium (§)	1.69 *** (0.55)	2.70 *** (0.59)
Perceived stress high (§)	1.47 *** (0.54)	2.30 *** (0.57)
Work Satisfaction in agriculture (1 to 5)	−0.31 (0.32)	−0.31 (0.30)
***Environmental***		
Urban (Dummy)	1.36 *** (0.50)	1.76 *** (0.45)
Constant	−5.64 ** (2.66)	−9.14 *** (2.62)
Observations	350	350

*** *p* < 0.01, ** *p* < 0.05, * *p* < 0.1, Standard errors in parentheses. Reference case(§): low perceived stress from heat and cold. Note: the number of implemented response measures were in response to either heat waves or cold spells, and the number of working days was either during the summer or winter, in the perceived productivity loss from the heat wave and cold spell models, respectively. The number of perceived events were in relation to either heat waves or cold spells, depending on the model. Numbers of perceived illnessses or symptoms were related to either heat or cold in the perceived productivity loss from the heat wave and cold spell models. Perceived stress medium and perceived stress high were also in response to either heat or cold with reference to low perceived stress in self-reported productivity loss from heat waves and cold spells.

## Supplementary Materials

The following are available online at https://www.mdpi.com/1660-4601/16/9/1578/s1, Table S1: Questions on perceived stress from heat and cold and associated productivity loss and health effects. Table S2: Results of ordered logit model with the dependent variables being the level of heat stress and cold stress (from 1 very low to 3 very high) by districts; Table S3: Determinants of self-reported labour productivity loss by districts; Table S4: Correlation matrix of determinants of the perceived level of heat stress (N = 350); Table S5: Correlation matrix of determinants of the perceived level of cold Stress (N = 350); Table S6: Correlations matrix of determinants of perceived labour productivity loss from heat waves (N = 350); Table S7: Correlations matrix of determinants of perceived labour productivity loss from cold stress (N = 350); Table S8: Impacts of the level of income and level of heat and cold stress on different coping strategies related to heat and cold by bivariate analysis(N = 350).
