# Supplementary material for: Socio-Economic Impact of and Adaptation to Extreme Heat and Cold of Farmers in the Food Bowl of Nepal
Source: Int J Environ Res Public Health. 2019 May 6;16(9):1578. doi: 10.3390/ijerph16091578 (PMC6539874; doi:10.3390/ijerph16091578)
Supplement: Supplementary file 1 [file ijerph-16-01578-s001.pdf]

Supplementary Materials

Table S1: Questions on perceived stress from heat and cold and associated productivity loss and health effects

Do you feel heat (cold) stressed during heat waves (cold spells) when you undertake your agricultural activities in a usual year during the last five years?	1. No, not at all<br>2. Yes, rarely<br>3. Sometimes<br>4. Often<br>5. Very often
If you felt heat (cold) stressed, did you find yourself, as a consequence, less productive when working on agriculture-related activities?	1. No, not at all<br>2. Yes, rarely<br>3. Sometimes<br>4. Often<br>5. Very often
Have heat waves/cold spells affected your health and the health of your family in the last five years?	1. Definitely yes<br>2. Probably yes<br>3. Probably not<br>4. Definitely not
If **yes,** how have heat waves/cold spells affected your and family's health over the last five years?	
Were there days in the last year when you could not work at all in the agricultural field because of extreme heat/cold?	1. Yes<br>2. No
If yes, how many days were you absent during extreme heat/cold?	
What preventative measures do you currently adopt to avoid heat/cold related stress in the agricultural field?	

Table S2: Results of ordered logit model with the dependent variables being the level of heat stress and cold stress (from 1 very low to 3 very high) by districts

	Perceived heat stress		Perceived cold stress	
Variables	Bardiya	Banke	Bardiya	Banke
Socio-economic				
Land size(In Bigga)	-0.05(0.12)	0.02(0.11)	-0.04(0.11)	-0.03(0.12)
Annual income(1-5)	0.33**(0.16)	0.08(0.14)	-0.04(0.19)	0.23(0.15)
Having access to weather information	-1.9***(0.4)	-0.51(0.37)	-1.09**(0.43)	-0.35(0.38)
Living in concrete or brick house	0.17(0.34)	-0.06(0.34)	0.24(0.32)	0.31(0.32)
Owning livestock	0.31(0.39)	0.59*(0.33)	0.60(0.39)	0.40(0.32)
Education (1 to 5)	0.47***(0.1)	-0.31*(0.16)	0.24(0.18)	0.09(0.16)
Physical				
Age	0.20***(0.08)	0.05(0.08)	0.16**(0.07)	0.02(0.07)
Age Square	-0.001**(0.00)	-0.00(0.001)	-0.001**(0.00)	-0.001(0.00)
Active family members(15-59 years)	0.01(0.07)	-0.01(0.05)	0.02(0.06)	-0.05(0.05)
Male	-0.18(0.39)	-0.10(0.38)	-0.11(0.37)	0.03(0.34)
Health status(1 to 3)	0.16(0.36)	-0.63**(0.29)	0.28(0.32)	0.07(0.27)
Implemented response measures	0.79** (0.32)	0.24(0.17)	0.97***(0.28)	0.37*(0.19)
Working days	0.01(0.01)	0.01**(0.01)	0.00(0.01)	0.00(0.01)
Psychological				
Perceived events(1 to 3)	0.24(0.43)	1.11***(0.25)	0.42*(0.26)	0.46**(0.19)
Health Satisfaction(1 to 3)	0.22(0.26)	0.42(0.26)	0.24(0.26)	-0.03(0.24)
Observations	167	183	167	183

*** p<0.01, ** p<0.05, * p<0.1; Standard errors in parentheses, [1] I Bigha = 0.67 ha. Note: the number of implemented response measures were either in response to heat waves or cold spells, and the number of working days was either during the summer or winter in the heat wave and cold spell model, respectively. The number of perceived events were either in relation to heat waves or cold spells, depending on the model.

Table S3: Determinants of self-reported labour productivity loss by districts

	Perceived labour productivity loss during heat waves		Perceived labour productivity loss during cold spells	
**Variables**	Bardiya	Banke	Bardiya	Banke
Socio-economic				
Land size (in Bigga)	–0.66 (0.42)	–0.12 (0.25)	–0.37 (0.29)	0.05 (0.18)
Annual income (1 to 5)	0.52 (0.69)	0.35 (0.24)	0.16 (0.50)	0.63*** (0.23)
Access to weather information	4.69 (3.57)	2.65*** (0.79)	2.1 (0.28)	2.56*** (0.68)
Living in concrete or brick house	1.17 (1.65)	0.44 (0.52)	0.01 (0.99)	0.60 (0.48)
Owning livestock	–2.63 (2.17)	0.85 (0.52)	–2.76 (1.75)	0.50 (0.46)
Education (1 to 5)	0.45 (0.87)	0.16 (0.24)	1.44* (0.80)	0.08 (0.23)
Physical				
Age	0.37 (0.28)	–0.05 (0.14)	0.55** (0.25)	0.20* (0.11)
Age square	–0.01* (0.00)	0.00 (0.00)	–0.01** (0.00)	–0.00* (0.00)
Active family members(15–59 years)	0.25 (0.29)	–0.07 (0.08)	0.02 (0.19)	–0.08 (0.07)
Male	0.72 (1.75)	–0.68 (0.57)	–1.91 (1.29)	–0.83 (0.54)
Health status (1 to 3)	–1.39 (1.57)	–0.13 (0.42)	1.78 (1.11)	–0.20 (0.38)
Perceived illnesses/symptoms	–0.43 (0.55)	0.54*** (0.19)	0.80 (0.74)	0.40 (0.27)
Implemented response measures	0.19 (1.07)	0.88*** (0.27)	0.46 (0.56)	0.40 (0.30)
Working days	–0.02 (0.04)	0.01 (0.01)	0.03 (0.03)	–0.01 (0.01)
Psychological				
Perceived events(1 to 3)	4.18** (1.72)	–0.05 (0.43)	0.08 (0.72)	0.13 (0.29)
Perceived stress medium(§)	3.24* (1.85)	1.93*** (0.71)	1.67 (1.43)	2.66*** (0.66)
Perceived stress high (§)	2.84 (1.76)	1.57** (0.66)	3.26** (1.50)	1.62** (0.64)
Work satisfaction in agriculture (1 to 5)	–1.01 (1.95)	–0.26 (0.36)	0.18 (1.06)	–0.29 (0.33)
Constant	–1.15 (10.78)	–4.39 (3.25)	–18.61** (9.47)	–8.18*** (2.95)
Observations	167	183	167	183

*** p<0.01, ** p<0.05, * p<0.1, Standard errors in parentheses. Reference case(§): low perceived stress from heat and cold. Note: the number of implemented response measures were in response to either heat waves or cold spells, and the number of working days was during either the summer or winter, in the perceived productivity loss from the heat wave and cold spell models, respectively. The number of perceived events were in relation to either heat waves or cold spells, depending on the model. Numbers of perceived illnesses or symptoms were related to either heat or cold in the perceived productivity loss from the heat wave and cold spell models. Perceived stress medium and perceived stress high were also in response to either heat or cold with reference to low perceived stress in self-reported productivity loss from heat waves and cold spells.

Table S4: Correlation matrix of determinants of the perceived level of heat stress (N = 350)

	Land	Income	Met	House	Livestock	Edu	Age	Active	Sex	Health	Cope	Days	PerC	Health PerC
**Income**	0.166	1												
**Met**	0.099	0.262	1											
**House**	–0.12	–0.177	0.099	1										
**Livestock**	0.178	0.077	–0.05	–0.02	1									
**Edu**	0.256	0.218	0.283	–0.06	–0.03	1								
**Age**	–0.08	–0.091	–0.145	0.052	0.102	–0.49	1							
**Active**	0.548	0.236	0.062	0.059	0.224	0.165	–0.03	1						
**Sex**	0.12	–0.024	–0.016	0.03	0.078	0.128	0.301	0.172	1					
**Health**	0.122	0.057	0.288	0.08	0.046	0.174	–0.14	0.035	–0.00	1				
**Cope**	0.095	0.204	0.104	–0.05	0.08	0.113	–0.08	–0.087	–0.16	0.307	1			
**Days**	0.114	–0.255	–0.061	0.09	0.081	–0.14	0.074	0.083	0.094	–0.069	–0.35	1		
**PerC**	–0.01	0.057	–0.071	–0.05	0.06	0.027	0	–0.016	–0.00	–0.036	0.009	–0.012	1	
**Health PerC**	0.032	–0.063	0.072	0.073	0.039	0.095	–0.12	0.056	0.097	0.287	0.098	–0.021	–0.12	1
**Urban**	0.12	0.169	0.123	–0.18	0.129	0.123	–0.11	–0.06	–0.14	0.124	0.335	–0.097	0.151	–0.019

Note: Land—household land size; Income—annual household income; Met—access to weather information; House—type of house; Livestock—having livestock or not; Edu—household head's level of education; Age—age of household's head; Active—total households members (aged between 15–59); Sex—sex of the household's head; Health—existing health status; Cope—numbers of implemented heat wave/cold spell adaptation measures; Days—number of working days during summer/winter seasons; PerC—perception of heat waves/cold spells; Stress—perceived level of heat (cold) stress during summer (winter); Work—level of work satisfaction working in agriculture; Health PerC—number of heat-/cold-related illnesses; Urban—urban or rural areas.

Table S5: Correlation matrix of determinants of the perceived level of cold Stress (N = 350)

	Land	Income	Met	House	Livestock	Edu	Age	Active	Sex	Health	Cope	Work	PerC	Health PerC
**Income**	0.166	1												
**Met**	0.099	0.262	1											
**House**	–0.12	–0.177	0.099	1										
**Livestock**	0.178	0.077	–0.05	–0.02	1									
**Edu**	0.256	0.218	0.283	–0.06	–0.03	1								
**Age**	–0.08	–0.091	–0.145	0.052	0.102	–0.49	1							
**Active**	0.548	0.236	0.062	0.059	0.224	0.165	–0.032	1						
**Sex**	0.12	–0.024	–0.016	0.03	0.078	0.128	0.301	0.172	1					
**Health**	0.122	0.057	0.288	0.08	0.046	0.174	–0.14	0.035	–0.00	1				
**Cope**	0.032	0.401	–0.061	–0.11	0.123	0.175	–0.07	0.021	–0.12	0.013	1			
**Work**	0.101	–0.399	–0.033	0.212	0.001	–0.07	0.095	0.057	0.148	–0.043	–0.56	1		
**PerC**	–0.06	0.404	0.296	–0.02	–0.067	–0.01	0.027	0.026	0.01	–0.128	0.157	–0.266	1	
**Health PerC**	0.032	–0.063	0.072	0.073	0.039	0.095	–0.126	0.056	0.097	0.287	–0.02	–0.063	–0.09	1
**Urban**	0.12	0.169	0.123	–0.18	0.129	0.123	–0.114	–0.068	–0.14	0.124	0.155	–0.193	–0.04	–0.019

Note: Land—household land size; Income—annual household income; Met—access to weather information; House—type of house; Livestock—having livestock or not; Edu—household head’s level of education; Age—age of household’s head; Active—total households members (aged between 15–59); Sex—sex of the household’s head; Health—existing health status; Cope—numbers of implemented heat wave/cold spell adaptation measures; Days—number of working days during summer/winter seasons; PerC—perception of heat waves/cold spells; Stress—perceived level of heat (cold) stress during summer (winter); Work—level of work satisfaction working in agriculture; Health PerC—number of heat-/cold-related illnesses; Urban—urban or rural areas.

Table S6: Correlations matrix of determinants of perceived labour productivity loss from heat waves (N = 350)

	Land	Income	Met	House	Livestock	Edu	Age	Active	Sex	Health	Health PerC	Cope	Days	PerC	Stress	Work
**Income**	0.16	1														
**Met**	0.09	0.26	1													
**House**	–0.12	–0.17	0.09	1												
**Livestock**	0.17	0.07	–0.05	–0.02	1											
**Edu**	0.25	0.21	0.28	–0.06	–0.03	1										
**Age**	–0.08	–0.09	–0.1	0.05	0.1	–0.49	1									
**Active**	0.54	0.23	0.06	0.05	0.22	0.16	–0.03	1								
**Sex**	0.12	–0.02	–0.01	0.03	0.07	0.12	0.3	0.17	1							
**Health**	0.12	0.05	0.28	0.08	0.04	0.17	–0.14	0.03	–0.007	1						
**Health PerC**	0.04	0.35	0.08	–0.16	0.12	0.1	–0.07	0.06	–0.1	0.04	1					
**Cope**	0.09	0.2	0.1	–0.05	0.08	0.11	–0.08	–0.08	–0.16	0.3	0.4	1				
**Days**	0.11	–0.25	–0.06	0.09	0.08	–0.14	0.07	0.08	0.09	–0.06	–0.36	–0.35	1			
**PerC**	–0.01	0.05	–0.07	–0.05	0.06	0.02	–0.006	–0.01	–0.004	–0.03	0.15	0.009	–0.01	1		
**Stress**	0.01	0.008	–0.24	–0.07	0.17	–0.07	0.16	0.02	0.03	–0.07	0.15	0.1	0.06	0.2	1	
**Work**	0.04	–0.04	0.02	0.07	0.05	–0.08	0.004	0.04	0.01	0.08	–0.08	0.009	0.11	–0.17	–0.04	1
**Urban**	0.12	0.16	0.12	–0.18	0.12	0.12	–0.11	–0.6	–0.14	0.12	0.31	0.33	–0.09	0.15	0.06	–0.13

Note: Land—household land size; Income—annual household income; Met—access to weather information; House—type of house; Livestock—having livestock or not; Edu—household head's level of education; Age—age of household's head; Active—total households members (aged between 15–59); Sex—sex of the household's head; Health—existing health status; Cope—numbers of implemented heat wave/cold spell adaptation measures; Days—number of working days during summer/winter seasons; PerC—perception of heat waves/cold spells; Stress—

perceived level of heat (cold) stress during summer (winter); Work—level of work satisfaction working in agriculture; Health PerC—number of heat-/cold-related illnesses; Urban—urban or rural areas.

Table S7: Correlations matrix of determinants of perceived labour productivity loss from cold stress (N = 350)

	**Land**	**Income**	**Met**	**House**	**Livestock**	**Edu**	**Age**	**Active**	**Sex**	**Health**	**Health PerC**	**Cope**	**Days**	**PerC**	**Stress**	**Work**
**Income**	0.16	1														
**Met**	0.09	0.26	1													
**House**	–0.12	–0.17	0.09	1												
**Livestock**	0.17	0.07	–0.05	–0.02	1											
**Edu**	0.25	0.21	0.28	–0.06	–0.03	1										
**Age**	–0.08	–0.09	–0.14	0.05	0.102	–0.49	1									
**Active**	0.54	0.23	0.06	0.05	0.22	0.16	–0.03	1								
**Sex**	0.12	–0.02	–0.01	0.03	0.07	0.12	0.3	0.17	1							
**Health**	0.12	0.05	0.28	0.08	0.04	0.17	–0.14	0.03	–0.01	1						
**Health PerC**	0.01	0.21	0.22	–0.16	0	0.06	–0.1	–0.08	–0.19	0.11	1					
**Cope**	0.03	0.4	–0.06	–0.11	0.12	0.17	–0.07	0.02	–0.12	0.01	0.36	1				
**Days**	0.1	–0.39	–0.03	0.21	0	–0.07	0.09	0.05	0.14	–0.04	–0.5	–0.56	1			

**PerC**	–0.06	0.4	0.29	–0.02	–0.06	–0.01	0.02	0.02	0.01	–0.18	0.16	0.15	–0.26	1		
**Stress**	0.03	0.18	–0.07	–0.04	0.17	0.06	0.04	0.002	–0.01	0.04	0.12	0.34	–0.22	0.11	1	
**Work**	0.04	–0.04	0.02	0.07	0.05	–0.01	0.004	0.04	0.01	0.08	–0.12	–0.04	0.12	–0.19	–0.1	1
**Urban**	0.12	0.16	0.12	–0.18	0.12	0.12	–0.11	–0.06	–0.14	0.12	0.35	0.15	–0.19	–0.04	0.21	–0.13

Note: Land—household land size; Income—annual household income; Met—access to weather information; House—type of house; Livestock—having livestock or not; Edu—household head's level of education; Age—age of household's head; Active—total households members (aged between 15–59); Sex—sex of the household's head; Health—existing health status; Cope—numbers of implemented heat wave/cold spell adaptation measures; Days—number of working days during summer/winter seasons; PerC—perception of heat waves/cold spells; Stress—perceived level of heat (cold) stress during summer (winter); Work—level of work satisfaction working in agriculture; Health PerC—number of heat-/cold-related illnesses; Urban—urban or rural areas.

Table S8: Impacts of the level of income and level of heat and cold stress on different coping strategies related to heat and cold by bivariate analysis(N = 350)

	**Impact on heat wave response strategies**				
	Hats/umbrella	Resting in shade/slowing down work	Stopping work	Reschedule working times	Cooling techniques
Level of perceived heat stress (1 to 3)	$\chi^2(2) = 0.049$<br>$p = 0.9758$	$\chi^2(2) = 0.007$<br>$p = 0.9970$	$\chi^2(2) = 5.035$<br>$p = 0.0807$	$\chi^2(2) = 10.39$<br>$p = 0.0055$	$\chi^2(2) = 1.801$<br>$p = 0.4064$
Annual household's income (1 to 5)	$\chi^2(4) = 24.82$<br>$p = 0.0001$	$\chi^2(4) = 9.282$<br>$p = 0.0544$	$\chi^2(4) = 46.77$<br>$p = 0.0001$	$\chi^2(4) = 9.968$<br>$p = 0.0426$	$\chi^2(4) = 18.15$<br>$p = 0.0012$
	**Impact on cold spells response strategies**				
	Warm clothes	Stopping work/ resting to warm up	Reschedule working times	Drinking hot	Others
Level of perceived cold stress (1 to 3)	$\chi^2(2) = 2.088$<br>$p = 0.3520$	$\chi^2(2) = 30.56$<br>$p = 0.0001$	$\chi^2(2) = 7.556$<br>$p = 0.0229$	$\chi^2(2) = 75.35$<br>$p = 0.0001$	
Annual household's income (NRP) (1 to 5)	$\chi^2(4) = 0.913$<br>$p = 0.9227$	$\chi^2(4) = 40.50$<br>$p = 0.001$	$\chi^2(4) =61.94$<br>$p = 0.001$	$\chi^2(4) = 59.06$<br>$p = 0.0001$	
